# Ice‐age persistence and genetic isolation of the disjunct distribution of larch in Alaska

**DOI:** 10.1002/ece3.6031

**Published:** 2020-01-23

**Authors:** Joseph D. Napier, Matias C. Fernandez, Guillaume de Lafontaine, Feng Sheng Hu

**Affiliations:** ^1^ Department of Plant Biology University of Illinois Urbana IL USA; ^2^ Department of Integrative Biology The University of Texas at Austin Austin TX USA; ^3^ Canada Research Chair in Integrative Biology of Northern Flora Université du Québec à Rimouski Rimouski QC Canada; ^4^ Department of Geology University of Illinois Urbana IL USA

**Keywords:** Alaska, climate relict, *Larix laricina*, last glacial maximum, range disjunction, refugia

## Abstract

*Larix laricina* (eastern larch, tamarack) is a transcontinental North American conifer with a prominent disjunction in the Yukon isolating the Alaskan distribution from the rest of its range. We investigate whether in situ persistence during the last glacial maximum (LGM) or long‐distance postglacial migration from south of the ice sheets resulted in the modern‐day Alaskan distribution. We analyzed variation in three chloroplast DNA regions of 840 trees from a total of 69 populations (24 new sampling sites situated on both sides of the Yukon range disjunction pooled with 45 populations from a published source) and conducted ensemble species distribution modeling (SDM) throughout Canada and United States to hindcast the potential range of *L. laricina* during the LGM. We uncovered the genetic signature of a long‐term isolation of larch populations in Alaska, identifying three endemic chlorotypes and low levels of genetic diversity. Range‐wide analysis across North America revealed the presence of a distinct Alaskan lineage. Postglacial gene flow across the Yukon divide was unidirectional, from Alaska toward previously glaciated Canadian regions, and with no evidence of immigration into Alaska. Hindcast SDM indicates one of the broadest areas of past climate suitability for *L. laricina* existed in central Alaska, suggesting possible in situ persistence of larch in Alaska during the LGM. Our results provide the first unambiguous evidence for the long‐term isolation of *L. laricina* in Alaska that extends beyond the last glacial period and into the present interglacial period. The lack of gene flow into Alaska along with the overall probability of larch occurrence in Alaska being currently lower than during the LGM suggests that modern‐day Alaskan larch populations are isolated climate relicts of broader glacial distributions, and so are particularly vulnerable to current warming trends.

## Graphical abstract

Range disjunctions represent natural laboratories to examine the interplay between long‐distance migration and refugial persistence in biome development. *Larix laricina* (larch) is a transcontinental North American conifer with a disjunction in the Yukon isolating its Alaskan distribution. Using chloroplast‐microsatellite analysis and species distribution modeling, we provide evidence for the glacial persistence and long‐term isolation of larch in Alaska.

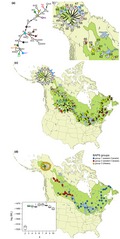

## 1 INTRODUCTION

With many ecosystems under threat from anthropogenic climate change, it is critical to understand species responses to past climate shifts in order to help project the impacts of future change (Blois, Zarnetske, Fitzpatrick, & Finnegan, 2013). The last glacial maximum (LGM; 23,000–19,000 cal yr BP) and its transition into the Holocene exhibited widespread changes in climate and species distributions (Jackson, Overpeck, Webb, Keattch, & Anderson, 1997; Williams, Shuman, Webb, Bartlein, & Leduc, 2004). Our understanding of the relative importance of ice‐age refugial persistence versus postglacial long‐distance migration in the development of modern species distributions has advanced greatly with recent integrative studies combining fossil, genetic, and modeling analyses (e.g., Gavin et al., 2014; Wang et al., 2015). These studies have called into question the role of long‐distance migration as the dominant species response to shifting climates (Christmas, Breed, & Lowe, 2016; Corlett & Westcott, 2013; de Lafontaine, Napier, Petit, & Hu, 2018). In particular, a growing number of phylogeographic surveys have demonstrated populations persisted in situ, in refugia closer to the ice sheets during the LGM than previously discernible from other lines of evidence (e.g., Anderson, Hu, Nelson, Petit, & Paige, 2006; de Lafontaine, Ducousso, Lefèvre, Magnanou, & Petit, 2013; McLachlan, Clark, & Manos, 2005; Napier, de Lafontaine, Heath, & Hu, 2019).

Range disjunctions serve as natural laboratories that allow us to examine the interplay between long‐distance migration and refugial persistence in biome development since the LGM (Bernardi, Findley, & Rocha‐Olivares, 2003; Givnish et al., 2004; Gusarova et al., 2015; Ruffley et al., 2018; Xiang & Soltis, 2001). Because range disjunctions limit gene flow and admixture, dispersal events from populations across a disjunction result in detectable genetic signals (Hamilton & Eckert, 2007; Sanz, Schönswetter, Vallès, Schneeweiss, & Vilatersana, 2014). Genetic analyses of disjunct populations from a number of species, such as *Pinus nigra* in western Europe, have highlighted the role of LGM refugia in postglacial colonization (e.g., Afzal‐Rafii & Dodd, 2007). These analyses also revealed complex postglacial population dynamics, uncovering the interplay between refugial persistence and long‐distance migration (Escudero, Valcárcel, Vargas, & Luceño, 2010; Fernandez, Hu, Gavin, de Lafontaine, & Heath, 2020; Wolf, Schneider, & Ranker, 2001). For example, although refugial populations of *Thuja plicata* persisted through the LGM in the interior of the Pacific Northwest, long‐distance immigrants from coastal populations dominated the interior range expansion during the postglacial period (Fernandez et al., 2020).

Tamarack (eastern larch, *Larix laricina*) is a transcontinental North American conifer, with a prominent disjunction in the Yukon that isolates the Alaskan distribution from the larger range in Canada (Ritchie, 1987; Warren et al., 2016). The isolated distribution in Alaska may have persisted through the LGM, or resulted from postglacial colonization and long‐distance migration from south of the ice sheets. Previous studies have provided evidence for the persistence of several boreal tree species through the LGM in ice‐free areas of Alaska and adjacent Canada (Anderson et al., 2006; Brubaker, Anderson, Edwards, & Lozhkin, 2005; Edwards, Armbruster, & Elias, 2014; Gerardi, Jaramillo‐Correa, Beaulieu, & Bousquet, 2010; Zazula, Telka, Harington, Schweger, & Mathewes, 2006). Little is known about the history of *L. laricina* despite several decades of paleoecological studies (Brubaker et al., 2005). Pollen records of the species are ambiguous but suggest the presence in parts of Alaska at least as early as 14 ka (Brubaker et al., 2005). A recent phylogeographic survey of *L. laricina*, which focused on the eastern part of the range, also indicated possible LGM persistence of the species in Alaska (Warren et al., 2016). However, this inference relied on a single genetically distinct population from Alaska, providing insufficient data to elucidate the development of the larch range disjunction.

Here, we report the results of an integrative study relying on chloroplast‐marker analyses and species distribution modeling to assess the origin and spatiotemporal dynamics of the disjunct Alaskan larch distribution. Specifically, we tested whether larch persisted in Alaska during the LGM or went through postglacial recolonization via long‐distance migration from populations that persisted south of the ice sheets. Under the former scenario, we expected a distinct genetic lineage in Alaska along with a high modeled probability of past occurrence, whereas postglacial migration should result in similar genetic lineages on both sides of the Yukon divide and a low probability of larch presence in the area during the LGM. We sampled 24 *L. laricina* populations on both sides of the Yukon range disjunction, including populations from Alaska, Northwest Territories, and Alberta. We analyzed these new results in a range‐wide transcontinental context by pooling them with published genotypic data on *L. laricina*, which provided key information for deciphering the postglacial spatiotemporal dynamics of *L. laricina* in the western part of its range (Warren et al., 2016). We also conducted ensemble species distribution modeling (SDM) to hindcast the potential range of *L. laricina* during the LGM*.* Together, these results provide the first unambiguous evidence for the long‐term ice‐age isolation of this species in Alaska that extends prior to the last glacial period and into the present interglacial period.

## 2 MATERIALS AND METHODS

### 2.1 Sampling, DNA extraction, and genotyping

We sampled 251 *L. laricina* individuals from 24 sites in Alaska and western Canada (Table S1). At each site, foliar samples from individuals spaced >100 m apart were collected and dried in silica gel. For each individual, total DNA was extracted from 18 mg of homogenized tissue using Nucleospin 96 Plant II (Macherey‐Nagel Inc.). Previous categorization of polymorphism for chloroplast DNA (cpDNA) in *L. laricina* was conducted by examining simple sequence repeats (SSRs) with 20 primer pairs (Warren et al., 2016). Three polymorphic chloroplast microsatellite (cpSSR) loci were identified: *Pt26081*, *Pt30204*, and *Pt63718* (Vendramin, Lelli, Rossi, & Morgante, 1996). We targeted these three loci for genotyping using the corresponding fluorescently tagged forward and reverse primers (Table S2). Each PCR mix contained 1× reaction buffer, 1.5 mm MgCl_2_, 0.1 mm of each dNTP, 0.16 μM of each fluorescently labeled primer (Invitrogen, Carlsbad, CA, USA), 0.75 unit of Platinum *Taq* polymerase, and 10 ng of template DNA. Amplification was performed by implementing a 3‐step program: (a) a 2‐min denaturation at 94°C; (b) 35 cycles of 30‐s denaturation at 94°C, 30‐s annealing at 55°C, and 2‐min elongation at 72°C; and (c) a 5‐min final elongation at 72°C.

The three target cpSSR loci were amplified for all larch samples and migrated in an ABI 3730xl automated sequencer (Applied Biosystems) with 50‐cm capillary arrays using LIZ500 fluorophores (6‐FAM, VIC, NED) by the W.M. Keck Center at the University of Illinois (Urbana‐Champaign, IL, USA). Fragments were then scored using the GeneMapper program (Applied Biosystems). Chlorotypes were obtained by concatenating alleles obtained at the three cpSSR loci.

A previous study (Warren et al., 2016) used these same three cpSSR loci to explore genetic patterns for *L. laricina* across 589 individuals from 45 populations sampled across eastern and western Canada (Table S1). We combined our new genotyping results with these previous data, resulting in 840 genotyped individuals spanning the full range of *L. laricina*. The combined dataset provides an opportunity to examine range‐wide genetic variation, allowing us to contextualize the patterns of genetic diversity present in the disjunct Alaskan population.

### 2.2 Genetic diversity, population structure, and differentiation

Within‐population chlorotype diversity (*H*_S_), which is the equivalent to expected heterozygosity for diploid data (Weir, 1996), was calculated with FSTAT 2.9 (Goudet, 1995). We conducted a Bayesian analysis of the population structure across all 840 genotyped *L. laricina* individuals in BAPS 6.0 (Corander, Marttinen, Sirén, & Tang, 2008) using “spatial clustering of groups” that identifies genetically homogenous populations based on the cpDNA polymorphisms. BAPS assigns populations in a preset number of user‐defined groups (the *k*‐value) seeking to maximize the differentiation among groups by considering the number of requested groups and their allele frequencies as varying parameters. The optimal *k*‐value (i.e., number of groups) is determined by running 10 replicates of *k* = 2 through 10 and then selecting the scenario where *log* marginal likelihood first reaches a plateau. Between the identified groups, an analysis of molecular variance (AMOVA) and pairwise *F*_ST_ among populations were implemented in ARLEQUIN version 3.5 (Excoffier & Lischer, 2010) to detect patterns of genetic differentiation.

### 2.3 Species distribution modeling

Species distribution modeling (SDM) was used to simulate modern‐day potential range and hindcast the LGM distribution for *L. laricina*. Occurrence records used to inform the SDMs were generated from our sampling sites and herbaria records (see Figure S1 in Appendix S1). The final dataset used to build the SDMs comprised 341 occurrence records. Climate data were extracted from WorldClim (Hijmans, Cameron, Parra, Jones, & Jarvis, 2005) and selected for present and LGM paleo‐environments (see Note S1 in Appendix S1). SDMs were then generated with BIOMOD2 package version 3.3 (Thuiller, Georges, Engler, & Breiner, 2016) implemented in R 3.3.3 (R Core Team, 2017; see Note S2 in Appendix S1). We evaluated model performance based on true skill statistics (TSS; Allouche, Tsoar, & Kadmon, 2006) and the area under the receiver operating characteristic curve (AUC; Fawcett, 2006). TSS ranges from −1 to +1, where +1 indicates perfect agreement and values of zero or less indicate a performance no better than random (Allouche et al., 2006). TSS values ranging from 0.2 to 0.5 are considered poor, from 0.6 to 0.8 useful, and >0.8 good to excellent (Coetzee, Robertson, Erasmus, Rensburg, & Thuiller, 2009). Models with AUC values above 0.75 are considered potentially useful (Elith, 2002), a random prediction has an AUC of 0.5 on average, and a perfect prediction achieves the maximum possible AUC of +1. A unique ensemble SDM was computed from the 50 best SDMs out of 700 models (see Note S2 in Appendix S1) based on their TSS values. The final ensemble SDM was projected onto present climate layers to visualize modern‐day potential range and on LGM climate to hindcast paleodistribution of *L. laricina*. The sensitivity and specificity of the ensemble model represent the proportion of modern presence and absence correctly predicted, respectively.

## 3 RESULTS

### 3.1 Genetic results

**Figure 1:**
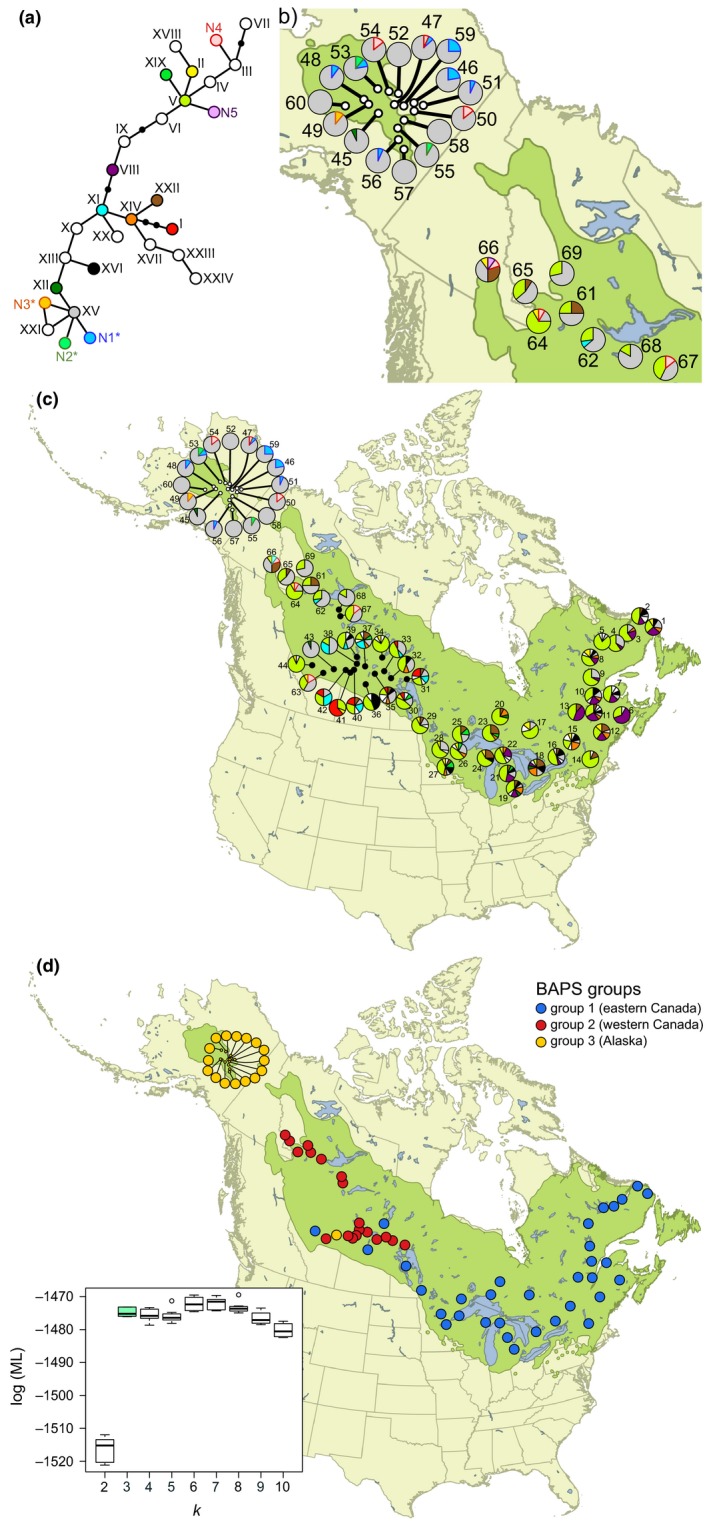
Patterns of genetic variation in three chloroplast DNA regions throughout the range of *Larix laricina* (species range shown in green). (a) Minimum spanning tree showing relationships among chlorotypes. Chlorotypes labeled in colored fonts represent new variants found in this study (*n* = 5), those identified with an asterisk are endemic to Alaska (*n* = 3), and those in white circles have a frequency below 0.01. (b, c) Geographic distribution of chlorotypes from populations sampled on either side of the Yukon range disjunction (b) and throughout the range (c). Population numbers correspond to those in Table S1. (d) Spatial Bayesian clustering of 69 *L. laricina* populations with an inset showing that the *log* marginal likelihood reaches a plateau when the number of groups (*k*) equals 3. Boxplots indicate lower quartile, median, and upper quartile over 10 replicate runs for each value of *k*; whisker length is 1.5 × interquartile range

**Figure 2:**
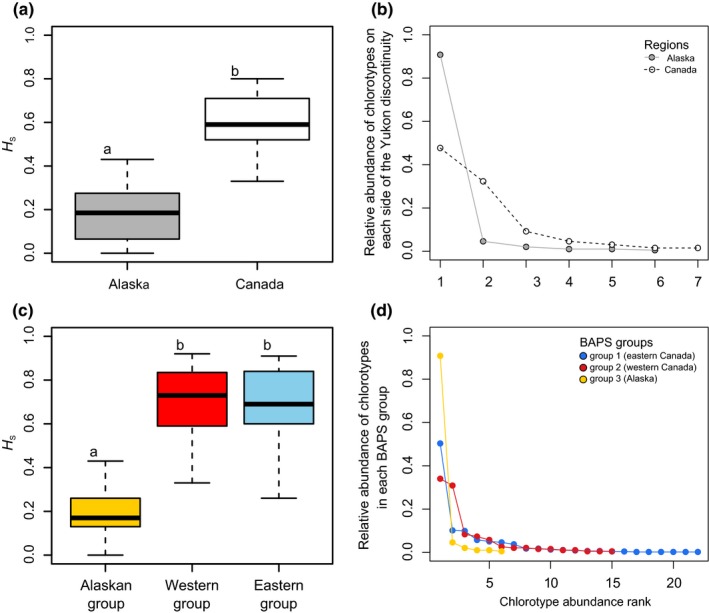
Comparisons of genetic diversity and evenness between Alaskan and Canadian populations located west and east of the Yukon discontinuity, respectively (a, b), and across the three BAPS groups (c, d). (a, c) Boxplots indicate lower quartile, median, and upper quartile of within‐population chlorotype diversity (*H*_S_), and whisker length is 1.5 × interquartile range. Different letters indicate statistically significant difference in genetic diversity. (b, d) Rank‐abundance curves of the chlorotypes. Steeper slopes indicate strong chlorotype dominance, and shallower gradients indicate that the relative abundance of chlorotypes is more similar

By concatenating the three polymorphic cpSSR loci, we identified a total of 29 multilocus chlorotypes over all sampled 840 larch individuals (Figure 1a; Table S1). Genetic analysis on the 24 new populations of *L. laricina* uncovered five previously unknown chlorotypes (N1–N5) of which three (N1, N2, and N3) are endemic to Alaska (Figure 1a,b). Larch samples focusing on both sides of the Yukon range discontinuity (Figure 1b; *n* = 24 populations) indicated that mean within‐population chlorotype diversity (*H*_S_) is significantly lower in Alaska compared with western Canadian populations east of the range discontinuity (Figure 2a; *p* = .0002, two‐sided Mann–Whitney U test). According to an analysis of molecular variance (AMOVA, Table 1a), 30% of the molecular variance in these populations is explained by grouping them geographically on either side of the Yukon discontinuity (*F*_CT_ = 0.3), whereas the molecular variance among the populations within these two regions is not statistically significant (*F*_SC_ = 0.04, *p* = .07). Alaskan populations exhibited dominance of a single chlorotype (XV), whereas greater evenness of the chlorotypes was found in Canada, across the disjunction (Figures 1b and 2b). The minimum spanning tree, which places chlorotypes with similar molecular variation close together and more differentiated chlorotypes further apart, identified that the three chlorotypes unique to Alaska are all one mutation away from the dominant chlorotype XV (Figure 1a).

**Table 1:** Analysis of molecular variance (AMOVA) that accounts for the sources of molecular variance in chlorotypes sampled throughout the range of *Larix laricina*

Source of variation	*df*	Var (%)	*F*‐statistics	*p*‐value
(a) Alaskan and Canadian populations located on either side of the Yukon range discontinuity (Figure 1b)				
Alaska versus Canada	1	30.14	*F*_CT_ = 0.301	<.001
Among populations within geographic region	22	2.92	*F*_SC_ = 0.042	.071
Within populations	228	66.94	*F*_ST_ = 0.331	<.001
Total	251			
(b) BAPS (Bayesian analysis of population structure) groups: Alaska, western Canada, and eastern Canada				
Among BAPS groups	2	29.48	*F*_CT_ = 0.295	<.001
Among populations within BAPS groups	66	3.90	*F*_SC_ = 0.055	<.001
Within populations	770	66.63	*F*_ST_ = 0.334	<.001
Total	838			

Abbreviations: *df*, degrees of freedom; Var (%), percentage of variation.

We merged our results with those of Warren et al. (2016) for the same species and cpDNA markers to examine the spatial patterns of genetic variation across North America (Figure 1c). The BAPS algorithm revealed three distinct population clusters (*log*ML of optimal partition = −1,473): group 1 in eastern Canada, group 2 in western Canada (from Lake Winnipeg to the Yukon range discontinuity), and group 3 in Alaska (Figure 1d, Table S1). The level of chlorotype diversity in the two Canadian BAPS groups is roughly equivalent (mean *H*_S_ = 0.68 and 0.71 for eastern and western Canada, respectively) but significantly higher than that of the Alaskan larch populations (Figure 2c, mean *H*_S_ = 0.19; *F* = 55.84, *p* < .0001, Tukey HSD). When compared to the Alaskan group, BAPS groups from Canada display greater evenness in the relative abundance of chlorotypes (Figures 1c and 2d). Chlorotypes V, VIII, XIV, XV, XVI, and XXIII are the most common in the east, whereas chlorotypes I, V, XI, XV, and XXII are dominant in western Canada (Figure 1c). The most common haplotype in Canada (V) was recovered in every population sampled east of the Yukon disjunction but was not found in Alaska (Figure 1c).

**Figure 3:**
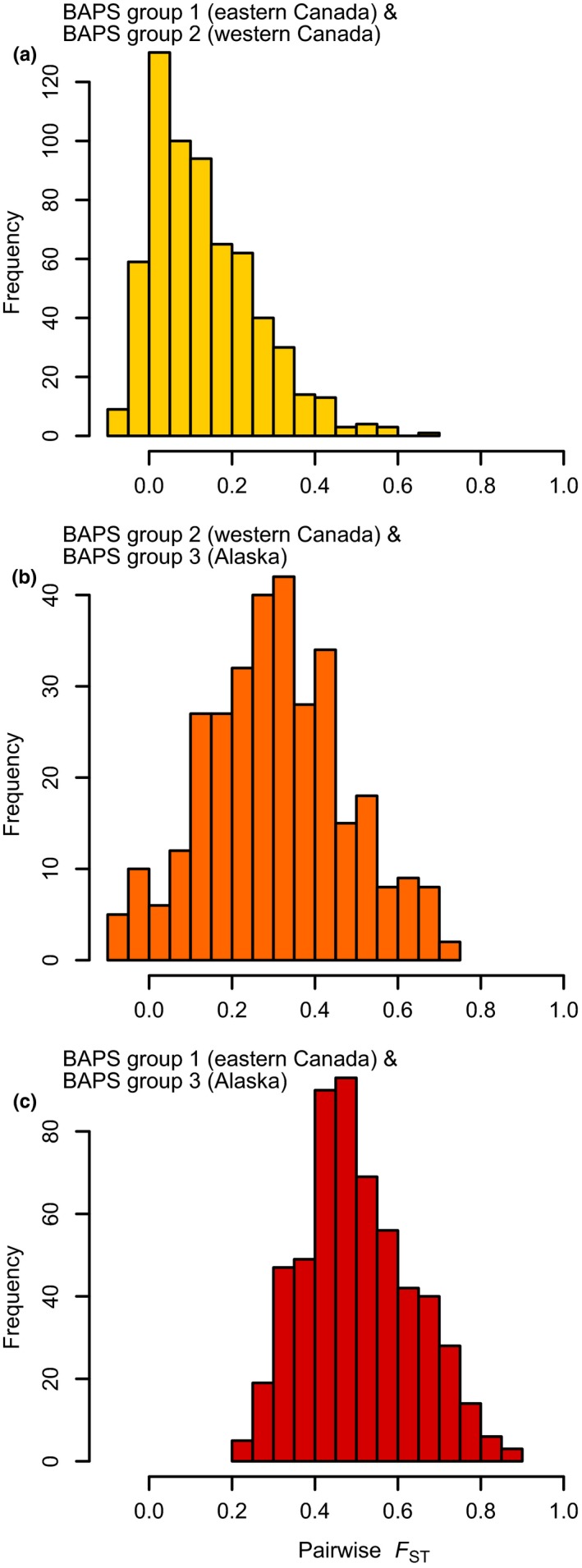
Frequency distribution of pairwise *F*_ST_ between populations from the two Canadian BAPS groups (a), the Alaskan group and the western Canadian group (b), and the Alaskan group and the eastern Canadian group (c)

Population differentiation among the three BAPS clusters varied greatly, as demonstrated by the distribution of pairwise *F*_ST_ between any two BAPS groups (Figure 3). The two BAPS groups from Canada are not highly differentiated (mean pairwise *F*_ST_ = 0.13, Figure 3a). Alaska populations are less differentiated from western Canadian populations (mean pairwise *F*_ST_ = 0.31, Figure 3b) than from the populations sampled in eastern Canada (mean pairwise *F*_ST_ = 0.50, Figure 3c). Analysis of molecular variance (AMOVA, Table 1b) showed that 30% of the molecular variance is accounted for by the three BAPS groups (*F*_CT_ = 0.295). A mere 4% of the variation is explained by populations within the BAPS groups (*F*_SC_ = 0.055), and the remaining 66% pertains to variance among individuals within the populations (*F*_ST_ = 0.334).

**Figure 4:**
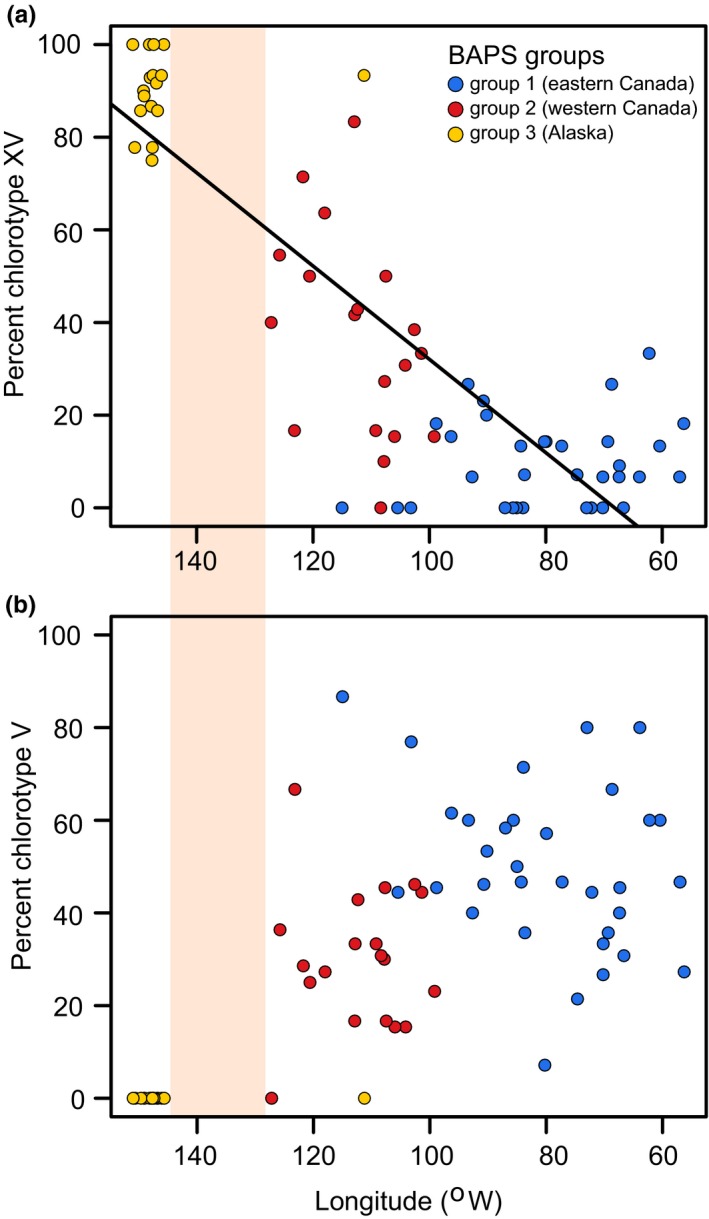
Longitudinal variation in the frequency of the most common chlorotypes found in *Larix laricina* populations sampled across North America. Circles of different colors indicate larch populations assigned to distinct BAPS groups. Orange shading illustrates the geographic position and extent of the Yukon range discontinuity

Chlorotypes XV and V are the two most common haplotypes found across the transcontinental range of tamarack, and their frequency distribution might provide insight on the direction and extent of postglacial gene flow (Figure 4). The relative abundance of chlorotype XV shows a steady decline from Alaska (90 ± 10%) toward eastern Canada where it stabilizes to 10 ± 9% (Figure 4a; *R* ^2^ = 0.7, *p* < .0001). Our data provide no evidence that chlorotype V ever crossed the Yukon discontinuity westward into Alaska, although the frequency of this haplotype remains high (42 ± 20%) across Canada and does not vary significantly along a longitudinal domain from 130°W to 55°W (Figure 4b; *R* ^2^ = .05, *p* = .07).

### 3.2 Species distribution models

**Figure 5:**
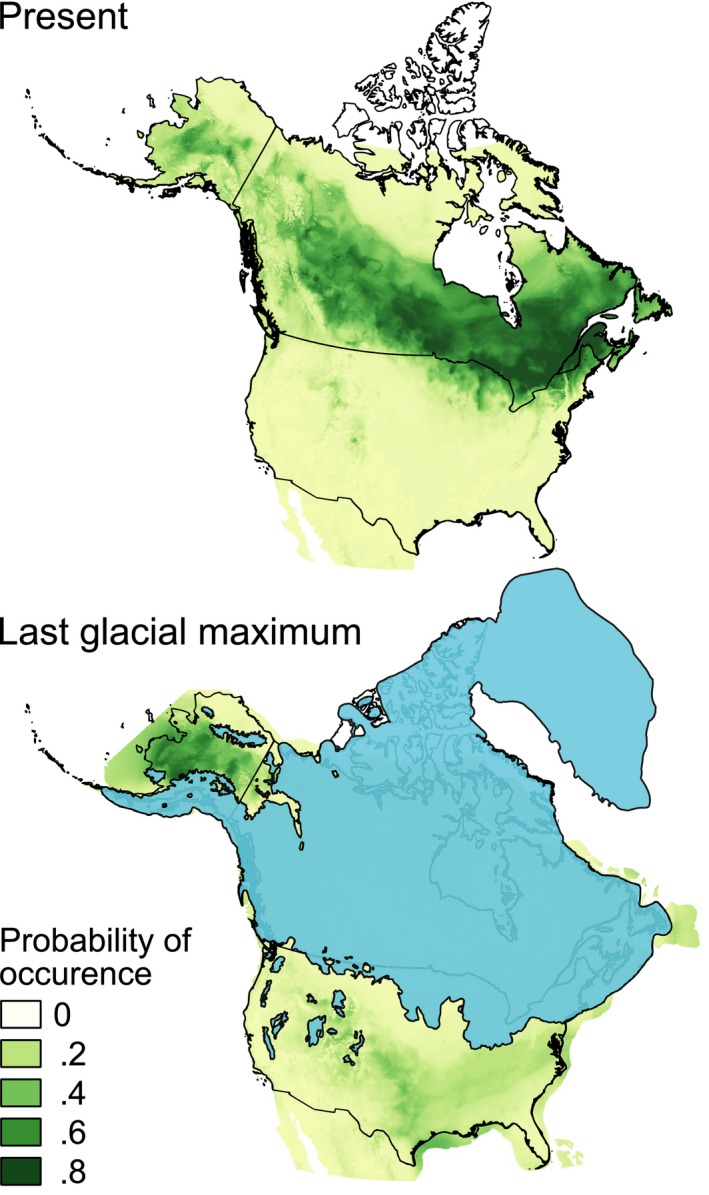
Ensemble species distribution models (SDMs) for *Larix laricina*. The top and lower panels show distributions predicted with the present and LGM (19–21 kyr ago) climatic conditions, respectively. Predicted distribution probabilities are shown in each 2.5 arc‐min pixel, with darker colors indicating increasing probability of occurrence. The blue layer represents the extent of the ice sheets during the last glacial maximum (Dyke, 2004)

Of the 700 models for *L. laricina*, 368 meet the criterion to be considered useful (TSS > 0.6)*.* We retained the 50 best models for the final ensemble SDM, with TSS values ranging between 0.70 and 0.81. The ensemble SDM for *L. laricina* predicts a modern distribution that matches well with the observed modern‐day range. The model suggests higher probability of occurrence in interior Alaska and throughout western and eastern Canada, and lower probability of presence in the United States (except for the Great Lake states and New England states where the species is indeed present), British Columbia, and Yukon (Figure 5). Various metrics (TSS = 0.709, AUC = 0.928, sensitivity = 95%, specificity = 75%) indicate that this ensemble SDM performs well and is thus useful for projecting the potential distribution of *L. laricina*. The predicted LGM distribution occurs in three distinct, isolated locations including modern Alaska and the Bering shelf, the Grand Banks of Newfoundland (the continental shelf far east of Canada), and south of the Laurentide ice sheet (Figure 5). Of these three locations, the largest continuous block harboring the highest suitable LGM habitats for larch occurs within the region that is now central Alaska.

## 4 DISCUSSION

Results from the 24 *L. laricina* populations revealed distinct genetic patterns on both sides of the Yukon range discontinuity. The populations from Alaska harbor three endemic chlorotypes (N1, N2, and N3), are largely dominated by a single chlorotype (XV; Figure 1), and seemingly display no evidence of incoming gene flow from Canadian populations (Figure 4b). The overall genetic diversity of the Alaskan group is significantly lower than what is found in populations directly across the disjunction as well as the primary Canadian distribution (Figure 2). The clear division of the *L. laricina* populations between eastern Canada, western Canada, and Alaska, as indicated by the cpDNA Bayesian analysis suggests the existence of three distinct ice‐age refugial lineages (Figure 1). These three glacial lineages are further supported by the presence of chlorotypes endemic to each BAPS group (Figure 1; Warren et al., 2016). The two lineages in eastern and western Canada were previously reported based on allozyme and cpDNA polymorphisms (Cheliak, Wang, & Pitel, 1988; Warren et al., 2016). Our results provide the first robust evidence for a third genetic lineage in Alaska, supporting prior speculation of a glacial refugium based upon the cpDNA analysis of a single Alaskan population (Warren et al., 2016).

The low genetic diversity of *L. laricina* in Alaska, especially when compared to the modern‐day conjoined populations forming the Canadian distribution, probably reflects long‐term isolation and erosion of genetic diversity due to drift (i.e., Frankham, 1996; Spielman, Brook, Briscoe, & Frankham, 2004; Willi, Buskirk, Schmid, & Fischer, 2007) fostered by the lack of incoming gene flow. Mutation rates in cpDNA and cpSSR loci are low in conifers (Provan, Powell, & Hollingsworth, 2001; Provan, Soranzo, Wilson, Goldstein, & Powell, 1999; Schaal & Olsen, 2000). For example, a conifer cpDNA study examining an 8,500‐year‐old bottleneck was unable to detect any mutations within 17 cpSSR loci and calculated that accumulating even a single mutation would likely require many additional generations (Provan et al., 1999). Thus, the endemic chlorotypes and multiple other chlorotypes that are one mutation step away from the most common chlorotype (XV) in Alaska strongly suggest prolonged isolation in the region (Figure 1). This geographic isolation likely predates the LGM, permitting little‐to‐no incoming gene flow from the more genetically diverse Canadian populations, promoting genetic drift in Alaska, and resulting in an increasingly divergent, albeit genetically depauperate, Alaskan lineage (de Lafontaine et al., 2013; Mee & Moore, 2014).

Our species distribution model corroborates the genetic data, supporting three LGM refugia for larch in North America (Figure 5). Furthermore, it suggests that one of the broadest areas of past climate suitability for *L. laricina* existed in central Alaska, which was unglaciated during the LGM (Kaufman & Manley, 2004). This bioclimatic scenario, combined with the withdrawal of many Alaskan competitor tree species into small refugial habitats during the LGM, would have provided larch with the potential opportunity to occupy a broader range than present. Evidence for such “reverse‐refugial dynamics” (i.e., ice‐age expansion rather than contraction) has been reported in a few other taxa during the LGM (Bisconti, Canestrelli, Colangelo, & Nascetti, 2011; Leite et al., 2016), but Alaskan larch would represent the first instance for a widespread boreal tree. It is possible that our SDM overestimated the extent of putative LGM distribution in Alaska, because of inaccurate paleoclimate data or the assumption of a static relationship between species and climate (Givnish et al., 2004; Guisan & Thuiller, 2005; Veloz et al., 2012). Additionally, abundant Alaskan fossil pollen records (Brubaker et al., 2005; Lozhkin & Anderson, 2011) do not provide evidence for pervasive larch persistence during the LGM and early postglacial. However, larch is extremely underrepresented in fossil pollen records, and sediment records spanning the LGM are rare (Brubaker et al., 2005), leaving open the possibility that a broad LGM distribution of *L. laricina* has been overlooked due to inadequate or missing data. Despite advances in arctoboreal palynology (Oswald, Brubaker, Hu, & Kling, 2003), pollen analysis will remain a blunt instrument for studying the LGM extent of larch in Alaska. Emerging techniques involving ancient plant DNA now allow for molecular reconstruction of palaeofloras through time (Parducci et al., 2017) and may provide an effective means to evaluate our results suggesting a broader past distribution of larch in Alaska during the LGM than at present.

Our results join a growing body of research demonstrating ice‐age persistence in areas previously thought to be climatically unsuitable for tree species during the LGM (e.g., Napier et al., 2019; Provan & Bennett, 2008; Roberts & Hamann, 2015; Stewart & Lister, 2001; Zeng, Wang, Liao, Wang, & Zhang, 2015). However, unlike other studies of boreal and temperate taxa that considered northern populations as “cryptic microrefugia” (Tzedakis, Emerson, & Hewitt, 2013), our results indicate that larch in Alaska during the LGM was more broadly distributed than present. Such a “reverse‐refugial dynamic” is thus in stark contrast with the LGM patterns generally reported for northern tree taxa and may have had an impact on the postglacial development of populations on both sides of the disjunction. Despite significant evidence of long‐term genetic isolation in Alaskan larch, our results also indicate a linear cline in the frequency of chlorotype XV that crosses the disjunction. The proportion of this chlorotype gradually declines with increasing distance from Alaska toward central Canada, suggesting substantial long‐distance gene flow of the Alaskan lineage (Figure 4). However, our results do not indicate any similar pattern of gene flow northward into Alaska. This asymmetrical pattern of gene flow (i.e., gene flow from Alaska into Canada but not vice versa) may have resulted from pollen swamping from Alaskan populations. The chloroplast genome is paternally inherited in many conifer taxa, including larch (Gros‐Louis, Bousquet, Pâques, & Isabel, 2005), and thus extensive pollen dispersal following the prevailing westerly winds from Alaska (Comtois, 1997) could have had a significant influence on the genetic composition of Canadian larch populations despite the presence of a range disjunction. As a lowland species, the Canadian Rocky Mountains present a persistent geographic barrier for larch expansion. Such a barrier, in combination with the prevailing wind direction, would have obstructed any larch migration while permitting only unidirectional pollen dispersal, consequently maintaining the genetic isolation of the Alaskan populations while still contributing to the pattern of southeastward gene flow seen in our data.

The proposed “reverse‐refugial dynamic” and asymmetrical gene flow may help explain the current distribution of Alaskan larch. In contrast with the broad habitat availability and larger effective population sizes during the LGM (Figure 5), the emergence of the current interglacial climate has trapped Alaskan larch populations within an isolated enclave of suitable habitats and rendered them glacial climate relicts (Hampe & Jump, 2011). The persistence of tree populations during periods of climate change depends upon their local adaptation or migration in response to the emerging environmental stresses (Aitken, Yeaman, Holliday, Wang, & Curtis‐McLane, 2008; de Lafontaine et al., 2018). The low genetic diversity in Alaskan larch populations (Figure 1) could reflect increased drift due to long‐term isolation and decreasing population sizes throughout the Holocene. With no “rescue effect” possible from external gene flow, an “Allee effect,” or decreased fitness associated with inbreeding depression in smaller population sizes, threatens the continued existence of these remnant populations (Stephens, Sutherland, & Freckleton, 1999). For instance, Alaskan larch has struggled to cope with cascading insect outbreaks, leading to the death of more than 80% of all adult larch in Alaska (Burnside, Schultz, Lisuzzo, & Kruse, 2013; Rozell, 2007). Given that, in addition to their genetic impoverishment, Alaskan larch populations are likely unable to migrate into new habitats, climate change may overwhelm their ability to persist. While there is an enduring debate over the preservation of peripheral populations, the longstanding isolation and endemic genetic signature of Alaskan larch suggests that they represent a unique ecological and evolutionary legacy worth conserving (Lesica & Allendorf, 1995). It may thus be prudent to sustain Alaskan larch populations through assisted migration to facilitate their movement across the disjunction before these climate relicts succumb to the mounting pressures of climate change.

## CONFLICT OF INTEREST

The authors have no conflict of interest to declare.

## AUTHOR CONTRIBUTIONS

F.S.H. and G.dL. designed the research. J.D.N. and G.dL. planned the fieldwork, while F.S.H. and G.dL. conducted it. J.D.N., M.C.F., and G.dL. performed laboratory work and analyzed data. All authors contributed to manuscript writing.

## Data Availability

Genetic data are available on Dryad repository (https://doi.org/10.5061/dryad.h9w0vt4dx).
