# Supplementary material for: Ice‐age persistence and genetic isolation of the disjunct distribution of larch in Alaska
Source: Ecol Evol. 2020 Jan 23;10(3):1692–702. doi: 10.1002/ece3.6031 (PMC7029070; doi:10.1002/ece3.6031)
Supplement: Supplementary file 1 [file ECE3-10-1692-s001.docx]

**Appendix S1**

**Note S1. WordClim bioclimatic data used in species distribution models**

In order to generate our species distribution models (SDMs), climate data needed to be selected for present and paleo environments. We extracted 19 bioclimatic WorldClim data layers (Hijmans, Cameron, Parra, Jones, & Jarvis, 2005; http://www.worldclim.org) at 2.5-arcmin resolution (~5 km at the equator) for the present (1960–2000) and the LGM (CMIP5 MIROC-ESM lgm; 21 kyr). Of all LGM paleoclimate models implemented in WorldClim, MIROC performs best for simulating precipitation and temperature in Alaska (Walsh, Chapman, Romanovsky, Christensen, & Stendel, 2008). Highly correlated climatic variables (r > 0.85) and those that contributed little to the predictive power (i.e., < 5% contribution) were removed from subsequent analyses. A set of six environmental predictors were used in our final SDM modeling (BIO1 = Annual Mean Temperature, BIO2 = Mean Diurnal Range, BIO4 = Temperature Seasonality, BIO5 = Max Temperature of Warmest Month, BIO11 = Mean Temperature of Coldest Quarter, and BIO14 = Precipitation of Driest Month).

**Note S2.** **Procedure for building species distribution models**

To generate our species distribution models (SDMs) within BIOMOD2, we used seven modeling algorithms: generalized linear models (GLM), boosted regression trees (GBM), artificial neural networks (ANN), flexible discriminant analysis (FDA), random forest (RF), classification tree analysis (CTA), and multivariate adaptive regression splines (MARS). For each model, the *Larix laricina* occurrence data were coupled with 500 pseudo-absences data generated randomly within the modeled study area (between longitudes 45 to 169°W and between latitudes 24 to 72°N) with equal weighting for presences and pseudo-absences (Barbet-Massin, Jiguet, Albert, & Thuiller, 2012). Models were trained with 80% of the coupled occurrences and pseudo-absence data and tested with the remaining 20%. Each modeling algorithm was run 100 times (i.e., 10 pseudo-absence samplings × 10 replicated runs), for a total of 700 models.

**Table S1.** Locations of 69 *Larix laricina* populations sampled for genetic analysis and number of each chlorotype per population.

| Site | Name | Source | BAPS | Sample | Latitude | Longitude | Chlorotype counts | | | | | | | | | | | | | | | | | | | | | | | | | | | | |
| --- | --- | --- | --- | --- | --- | --- | --- | --- | --- | --- | --- | --- | --- | --- | --- | --- | --- | --- | --- | --- | --- | --- | --- | --- | --- | --- | --- | --- | --- | --- | --- | --- | --- | --- | --- |
|  |  |  | group | size | (°N) | (°W) | I | II | III | IV | V | VI | VII | VIII | IX | X | XI | XII | XIII | XIV | XV | XVI | XVII | XVIII | XIX | XX | XXI | XXII | XXIII | XXIV | N1 | N2 | N3 | N4 | N5 |
| 1 | Port Hope Simpson | Warren et al. (2016) | 1 | 11 | 52.53 | 56.29 | 0 | 1 | 0 | 0 | 3 | 0 | 0 | 3 | 0 | 0 | 0 | 0 | 0 | 1 | 2 | 1 | 0 | 0 | 0 | 0 | 0 | 0 | 0 | 0 | 0 | 0 | 0 | 0 | 0 |
| 2 | Cartwright | Warren et al. (2016) | 1 | 15 | 53.71 | 57.00 | 0 | 0 | 0 | 0 | 7 | 0 | 0 | 2 | 2 | 0 | 0 | 0 | 0 | 0 | 1 | 2 | 0 | 0 | 0 | 0 | 0 | 1 | 0 | 0 | 0 | 0 | 0 | 0 | 0 |
| 3 | Goose River North | Warren et al. (2016) | 1 | 15 | 53.40 | 60.43 | 0 | 0 | 0 | 0 | 9 | 0 | 0 | 3 | 0 | 0 | 0 | 0 | 0 | 1 | 2 | 0 | 0 | 0 | 0 | 0 | 0 | 0 | 0 | 0 | 0 | 0 | 0 | 0 | 0 |
| 4 | Happy Valley | Warren et al. (2016) | 1 | 15 | 53.20 | 62.23 | 0 | 0 | 0 | 0 | 9 | 0 | 0 | 1 | 0 | 0 | 0 | 0 | 0 | 0 | 5 | 0 | 0 | 0 | 0 | 0 | 0 | 0 | 0 | 0 | 0 | 0 | 0 | 0 | 0 |
| 5 | Churchill Falls | Warren et al. (2016) | 1 | 15 | 53.56 | 63.91 | 0 | 0 | 0 | 1 | 12 | 0 | 0 | 0 | 0 | 0 | 0 | 0 | 0 | 0 | 1 | 0 | 1 | 0 | 0 | 0 | 0 | 0 | 0 | 0 | 0 | 0 | 0 | 0 | 0 |
| 6 | Stanley | Warren et al. (2016) | 1 | 13 | 46.27 | 66.65 | 0 | 0 | 0 | 0 | 4 | 0 | 0 | 8 | 1 | 0 | 0 | 0 | 0 | 0 | 0 | 0 | 0 | 0 | 0 | 0 | 0 | 0 | 0 | 0 | 0 | 0 | 0 | 0 | 0 |
| 7 | Amqui | Warren et al. (2016) | 1 | 11 | 48.44 | 67.35 | 0 | 0 | 0 | 0 | 5 | 0 | 0 | 2 | 0 | 0 | 0 | 0 | 0 | 0 | 1 | 1 | 1 | 0 | 0 | 0 | 0 | 0 | 0 | 1 | 0 | 0 | 0 | 0 | 0 |
| 8 | Fermont | Warren et al. (2016) | 1 | 15 | 52.79 | 67.40 | 0 | 2 | 1 | 0 | 6 | 0 | 0 | 2 | 0 | 0 | 0 | 0 | 0 | 1 | 1 | 1 | 0 | 0 | 0 | 0 | 1 | 0 | 0 | 0 | 0 | 0 | 0 | 0 | 0 |
| 9 | Manic 5 | Warren et al. (2016) | 1 | 15 | 50.66 | 68.68 | 0 | 0 | 0 | 0 | 10 | 0 | 0 | 1 | 0 | 0 | 0 | 0 | 0 | 0 | 4 | 0 | 0 | 0 | 0 | 0 | 0 | 0 | 0 | 0 | 0 | 0 | 0 | 0 | 0 |
| 10 | Reservoir Bersimis-2 | Warren et al. (2016) | 1 | 14 | 49.17 | 69.34 | 0 | 0 | 0 | 0 | 5 | 0 | 1 | 3 | 0 | 0 | 0 | 0 | 0 | 1 | 2 | 2 | 0 | 0 | 0 | 0 | 0 | 0 | 0 | 0 | 0 | 0 | 0 | 0 | 0 |
| 11 | La Malbaie | Warren et al. (2016) | 1 | 15 | 47.60 | 70.20 | 0 | 0 | 0 | 0 | 5 | 0 | 0 | 4 | 0 | 0 | 0 | 0 | 0 | 1 | 1 | 1 | 1 | 1 | 0 | 0 | 0 | 1 | 0 | 0 | 0 | 0 | 0 | 0 | 0 |
| 12 | Jackman | Warren et al. (2016) | 1 | 15 | 45.49 | 70.22 | 0 | 2 | 0 | 0 | 4 | 0 | 0 | 3 | 0 | 0 | 0 | 0 | 0 | 2 | 0 | 0 | 1 | 0 | 0 | 0 | 0 | 3 | 0 | 0 | 0 | 0 | 0 | 0 | 0 |
| 13 | Roberval | Warren et al. (2016) | 1 | 9 | 48.17 | 72.16 | 0 | 0 | 0 | 0 | 4 | 0 | 0 | 4 | 0 | 0 | 0 | 0 | 0 | 0 | 0 | 0 | 0 | 0 | 0 | 0 | 0 | 1 | 0 | 0 | 0 | 0 | 0 | 0 | 0 |
| 14 | South Wallingford | Warren et al. (2016) | 1 | 15 | 43.34 | 72.99 | 0 | 0 | 0 | 0 | 12 | 0 | 0 | 0 | 0 | 0 | 0 | 0 | 0 | 0 | 0 | 0 | 0 | 0 | 0 | 0 | 0 | 2 | 1 | 0 | 0 | 0 | 0 | 0 | 0 |
| 15 | Grenville Bay | Warren et al. (2016) | 1 | 14 | 45.65 | 74.63 | 0 | 1 | 0 | 2 | 3 | 0 | 0 | 0 | 0 | 0 | 0 | 0 | 1 | 3 | 1 | 2 | 1 | 0 | 0 | 0 | 0 | 0 | 0 | 0 | 0 | 0 | 0 | 0 | 0 |
| 16 | Elzevir | Warren et al. (2016) | 1 | 15 | 44.63 | 77.27 | 0 | 0 | 0 | 1 | 7 | 0 | 0 | 1 | 0 | 1 | 0 | 0 | 0 | 0 | 2 | 2 | 0 | 0 | 0 | 0 | 0 | 1 | 0 | 0 | 0 | 0 | 0 | 0 | 0 |
| 17 | Englehart | Warren et al. (2016) | 1 | 14 | 47.90 | 79.95 | 0 | 2 | 0 | 2 | 8 | 0 | 0 | 0 | 0 | 0 | 0 | 0 | 0 | 0 | 2 | 0 | 0 | 0 | 0 | 0 | 0 | 0 | 0 | 0 | 0 | 0 | 0 | 0 | 0 |
| 18 | Shelburne | Warren et al. (2016) | 1 | 14 | 44.07 | 80.24 | 0 | 0 | 0 | 0 | 1 | 1 | 0 | 1 | 0 | 0 | 0 | 1 | 0 | 3 | 2 | 2 | 0 | 0 | 0 | 0 | 0 | 3 | 0 | 0 | 0 | 0 | 0 | 0 | 0 |
| 19 | Holly | Warren et al. (2016) | 1 | 14 | 42.77 | 83.66 | 0 | 0 | 0 | 0 | 5 | 1 | 0 | 2 | 0 | 0 | 0 | 1 | 0 | 2 | 1 | 1 | 0 | 0 | 0 | 0 | 0 | 1 | 0 | 0 | 0 | 0 | 0 | 0 | 0 |
| 20 | Fushimi Lake Prov Park | Warren et al. (2016) | 1 | 14 | 49.84 | 83.92 | 0 | 0 | 0 | 0 | 10 | 0 | 0 | 0 | 0 | 0 | 0 | 1 | 0 | 2 | 0 | 0 | 0 | 0 | 0 | 0 | 0 | 1 | 0 | 0 | 0 | 0 | 0 | 0 | 0 |
| 21 | Tittabawassee River | Warren et al. (2016) | 1 | 15 | 44.09 | 84.29 | 0 | 0 | 0 | 0 | 7 | 0 | 0 | 3 | 0 | 0 | 0 | 0 | 0 | 0 | 2 | 1 | 0 | 0 | 1 | 0 | 0 | 1 | 0 | 0 | 0 | 0 | 0 | 0 | 0 |
| 22 | Wilderness State Park | Warren et al. (2016) | 1 | 14 | 45.70 | 85.01 | 0 | 1 | 0 | 0 | 7 | 1 | 1 | 3 | 0 | 0 | 0 | 0 | 0 | 1 | 0 | 0 | 0 | 0 | 0 | 0 | 0 | 0 | 0 | 0 | 0 | 0 | 0 | 0 | 0 |
| 23 | White Lake Prov Park | Warren et al. (2016) | 1 | 15 | 48.69 | 85.64 | 0 | 0 | 0 | 0 | 9 | 0 | 0 | 0 | 0 | 0 | 0 | 0 | 0 | 1 | 0 | 0 | 0 | 0 | 1 | 0 | 0 | 4 | 0 | 0 | 0 | 0 | 0 | 0 | 0 |
| 24 | Hiawatha Natl Forest | Warren et al. (2016) | 1 | 12 | 45.96 | 87.00 | 0 | 1 | 0 | 0 | 7 | 0 | 0 | 0 | 0 | 0 | 0 | 0 | 0 | 0 | 0 | 1 | 0 | 0 | 0 | 0 | 0 | 3 | 0 | 0 | 0 | 0 | 0 | 0 | 0 |
| 25 | Shebandowan | Warren et al. (2016) | 1 | 15 | 48.62 | 90.18 | 0 | 0 | 0 | 0 | 8 | 0 | 0 | 0 | 0 | 0 | 0 | 0 | 0 | 0 | 3 | 0 | 0 | 0 | 2 | 0 | 0 | 2 | 0 | 0 | 0 | 0 | 0 | 0 | 0 |
| 26 | Apostle Isl Natl Lakeshore | Warren et al. (2016) | 1 | 13 | 46.93 | 90.72 | 0 | 1 | 0 | 1 | 6 | 0 | 0 | 0 | 0 | 0 | 0 | 0 | 0 | 1 | 3 | 0 | 0 | 0 | 1 | 0 | 0 | 0 | 0 | 0 | 0 | 0 | 0 | 0 | 0 |
| 27 | Saint Croix State Forest | Warren et al. (2016) | 1 | 15 | 46.12 | 92.67 | 0 | 1 | 0 | 0 | 6 | 0 | 0 | 0 | 0 | 0 | 0 | 0 | 0 | 1 | 1 | 2 | 0 | 0 | 2 | 0 | 0 | 1 | 1 | 0 | 0 | 0 | 0 | 0 | 0 |
| 28 | Blackberry | Warren et al. (2016) | 1 | 15 | 47.22 | 93.36 | 0 | 1 | 0 | 0 | 9 | 1 | 0 | 0 | 0 | 0 | 0 | 0 | 0 | 0 | 4 | 0 | 0 | 0 | 0 | 0 | 0 | 0 | 0 | 0 | 0 | 0 | 0 | 0 | 0 |
| 29 | Richer | Warren et al. (2016) | 1 | 13 | 49.66 | 96.28 | 0 | 1 | 0 | 0 | 8 | 1 | 0 | 0 | 0 | 0 | 0 | 0 | 0 | 0 | 2 | 0 | 0 | 0 | 0 | 0 | 0 | 1 | 0 | 0 | 0 | 0 | 0 | 0 | 0 |
| 30 | Grahamdal | Warren et al. (2016) | 1 | 11 | 52.08 | 98.84 | 1 | 1 | 0 | 0 | 5 | 0 | 0 | 0 | 0 | 0 | 0 | 0 | 0 | 0 | 2 | 0 | 0 | 0 | 1 | 0 | 0 | 0 | 1 | 0 | 0 | 0 | 0 | 0 | 0 |
| 31 | Minago River | Warren et al. (2016) | 2 | 13 | 54.19 | 99.18 | 3 | 0 | 1 | 0 | 3 | 0 | 1 | 0 | 0 | 0 | 2 | 0 | 0 | 0 | 2 | 0 | 0 | 0 | 0 | 0 | 0 | 1 | 0 | 0 | 0 | 0 | 0 | 0 | 0 |
| 32 | Cranberry Portage | Warren et al. (2016) | 2 | 9 | 54.55 | 101.38 | 0 | 0 | 0 | 0 | 4 | 0 | 0 | 0 | 0 | 0 | 0 | 0 | 0 | 1 | 3 | 1 | 0 | 0 | 0 | 0 | 0 | 0 | 0 | 0 | 0 | 0 | 0 | 0 | 0 |
| 33 | Granit Lake | Warren et al. (2016) | 2 | 13 | 54.84 | 102.59 | 1 | 0 | 0 | 0 | 6 | 0 | 0 | 0 | 0 | 0 | 1 | 0 | 0 | 0 | 5 | 0 | 0 | 0 | 0 | 0 | 0 | 0 | 0 | 0 | 0 | 0 | 0 | 0 | 0 |
| 34 | Southend | Warren et al. (2016) | 1 | 13 | 56.16 | 103.20 | 0 | 1 | 1 | 0 | 10 | 0 | 0 | 0 | 0 | 0 | 0 | 1 | 0 | 0 | 0 | 0 | 0 | 0 | 0 | 0 | 0 | 0 | 0 | 0 | 0 | 0 | 0 | 0 | 0 |
| 35 | Big Sandy Lake | Warren et al. (2016) | 2 | 13 | 54.50 | 104.16 | 1 | 0 | 0 | 0 | 2 | 2 | 1 | 0 | 0 | 0 | 0 | 0 | 0 | 1 | 4 | 1 | 0 | 0 | 0 | 0 | 0 | 1 | 0 | 0 | 0 | 0 | 0 | 0 | 0 |
| 36 | Prince Albert | Warren et al. (2016) | 1 | 9 | 53.36 | 105.47 | 0 | 0 | 0 | 1 | 4 | 0 | 0 | 0 | 0 | 0 | 0 | 0 | 0 | 0 | 0 | 4 | 0 | 0 | 0 | 0 | 0 | 0 | 0 | 0 | 0 | 0 | 0 | 0 | 0 |
| 37 | Morin Lake | Warren et al. (2016) | 2 | 13 | 55.10 | 105.99 | 0 | 0 | 1 | 0 | 2 | 0 | 1 | 0 | 0 | 0 | 3 | 0 | 0 | 1 | 2 | 0 | 0 | 0 | 1 | 0 | 0 | 2 | 0 | 0 | 0 | 0 | 0 | 0 | 0 |
| 38 | Lac la Plonge | Warren et al. (2016) | 2 | 6 | 55.21 | 107.49 | 0 | 0 | 0 | 0 | 1 | 0 | 0 | 0 | 0 | 0 | 2 | 0 | 0 | 0 | 3 | 0 | 0 | 0 | 0 | 0 | 0 | 0 | 0 | 0 | 0 | 0 | 0 | 0 | 0 |
| 39 | Patuanak | Warren et al. (2016) | 2 | 11 | 55.88 | 107.70 | 0 | 0 | 0 | 0 | 5 | 0 | 0 | 0 | 0 | 0 | 1 | 0 | 0 | 0 | 3 | 1 | 0 | 0 | 0 | 1 | 0 | 0 | 0 | 0 | 0 | 0 | 0 | 0 | 0 |
| 40 | Aubichon Lake | Warren et al. (2016) | 2 | 10 | 54.59 | 107.82 | 2 | 0 | 0 | 0 | 3 | 0 | 1 | 0 | 0 | 0 | 2 | 0 | 0 | 0 | 1 | 0 | 0 | 0 | 0 | 0 | 0 | 1 | 0 | 0 | 0 | 0 | 0 | 0 | 0 |
| 41 | Meadow River | Warren et al. (2016) | 2 | 13 | 54.25 | 108.36 | 8 | 1 | 0 | 0 | 4 | 0 | 0 | 0 | 0 | 0 | 0 | 0 | 0 | 0 | 0 | 0 | 0 | 0 | 0 | 0 | 0 | 0 | 0 | 0 | 0 | 0 | 0 | 0 | 0 |
| 42 | Goodsoil | Warren et al. (2016) | 2 | 6 | 54.40 | 109.23 | 1 | 0 | 0 | 0 | 2 | 0 | 0 | 0 | 0 | 0 | 2 | 0 | 0 | 0 | 1 | 0 | 0 | 0 | 0 | 0 | 0 | 0 | 0 | 0 | 0 | 0 | 0 | 0 | 0 |
| 43 | Glendon | Warren et al. (2016) | 3 | 15 | 54.24 | 111.24 | 0 | 0 | 0 | 0 | 0 | 0 | 0 | 0 | 0 | 0 | 0 | 1 | 0 | 0 | 14 | 0 | 0 | 0 | 0 | 0 | 0 | 0 | 0 | 0 | 0 | 0 | 0 | 0 | 0 |
| 44 | Whitecourt | Warren et al. (2016) | 1 | 15 | 54.09 | 115.01 | 0 | 1 | 0 | 0 | 13 | 1 | 0 | 0 | 0 | 0 | 0 | 0 | 0 | 0 | 0 | 0 | 0 | 0 | 0 | 0 | 0 | 0 | 0 | 0 | 0 | 0 | 0 | 0 | 0 |
| 45 | Fairbanks | Warren et al. (2016) | 3 | 14 | 64.42 | 148.0 | 0 | 0 | 0 | 0 | 0 | 0 | 0 | 0 | 0 | 0 | 0 | 1 | 0 | 0 | 13 | 0 | 0 | 0 | 0 | 0 | 0 | 0 | 0 | 0 | 0 | 0 | 0 | 0 | 0 |
| 46 | AK01 | This study | 3 | 9 | 64.95 | 147.61 | 0 | 0 | 0 | 0 | 0 | 0 | 0 | 0 | 0 | 0 | 0 | 0 | 0 | 0 | 7 | 0 | 0 | 0 | 0 | 0 | 0 | 0 | 0 | 0 | 2 | 0 | 0 | 0 | 0 |
| 47 | AK02 | This study | 3 | 15 | 64.86 | 147.81 | 0 | 0 | 0 | 0 | 0 | 0 | 0 | 0 | 0 | 0 | 0 | 0 | 0 | 0 | 13 | 0 | 0 | 0 | 0 | 0 | 0 | 0 | 0 | 0 | 1 | 0 | 0 | 1 | 0 |
| 48 | AK03 | This study | 3 | 10 | 64.31 | 149.13 | 0 | 0 | 0 | 0 | 0 | 0 | 0 | 0 | 0 | 0 | 0 | 0 | 0 | 0 | 9 | 0 | 0 | 0 | 0 | 0 | 0 | 0 | 0 | 0 | 1 | 0 | 0 | 0 | 0 |
| 49 | AK04 | This study | 3 | 9 | 64.32 | 149.03 | 0 | 0 | 0 | 0 | 0 | 0 | 0 | 0 | 0 | 0 | 0 | 0 | 0 | 0 | 8 | 0 | 0 | 0 | 0 | 0 | 0 | 0 | 0 | 0 | 0 | 0 | 1 | 0 | 0 |
| 50 | AK05 | This study | 3 | 14 | 65.27 | 146.72 | 0 | 0 | 0 | 0 | 0 | 0 | 0 | 0 | 0 | 0 | 0 | 0 | 0 | 0 | 12 | 0 | 0 | 0 | 0 | 0 | 0 | 0 | 0 | 0 | 0 | 0 | 0 | 2 | 0 |
| 51 | AK06 | This study | 3 | 15 | 65.12 | 147.5 | 0 | 0 | 0 | 0 | 0 | 0 | 0 | 0 | 0 | 0 | 0 | 0 | 0 | 0 | 14 | 0 | 0 | 0 | 0 | 0 | 0 | 0 | 0 | 0 | 1 | 0 | 0 | 0 | 0 |
| 52 | AK07 | This study | 3 | 13 | 65.28 | 148.13 | 0 | 0 | 0 | 0 | 0 | 0 | 0 | 0 | 0 | 0 | 0 | 0 | 0 | 0 | 13 | 0 | 0 | 0 | 0 | 0 | 0 | 0 | 0 | 0 | 0 | 0 | 0 | 0 | 0 |
| 53 | AK08 | This study | 3 | 9 | 64.99 | 150.64 | 0 | 0 | 0 | 0 | 0 | 0 | 0 | 0 | 0 | 0 | 0 | 0 | 0 | 0 | 7 | 0 | 0 | 0 | 0 | 0 | 0 | 0 | 0 | 0 | 1 | 1 | 0 | 0 | 0 |
| 54 | AK09 | This study | 3 | 7 | 65.21 | 149.64 | 0 | 0 | 0 | 0 | 0 | 0 | 0 | 0 | 0 | 0 | 0 | 0 | 0 | 0 | 6 | 0 | 0 | 0 | 0 | 0 | 0 | 0 | 0 | 0 | 0 | 0 | 0 | 1 | 0 |
| 55 | AK10 | This study | 3 | 12 | 64.46 | 146.92 | 0 | 0 | 0 | 0 | 0 | 0 | 0 | 0 | 0 | 0 | 0 | 0 | 0 | 0 | 11 | 0 | 0 | 0 | 0 | 0 | 0 | 0 | 0 | 0 | 0 | 1 | 0 | 0 | 0 |
| 56 | AK11 | This study | 3 | 15 | 64.24 | 146.05 | 0 | 0 | 0 | 0 | 0 | 0 | 0 | 0 | 0 | 0 | 0 | 0 | 0 | 0 | 14 | 0 | 0 | 0 | 0 | 0 | 0 | 0 | 0 | 0 | 1 | 0 | 0 | 0 | 0 |
| 57 | AK12 | This study | 3 | 11 | 64.01 | 145.65 | 0 | 0 | 0 | 0 | 0 | 0 | 0 | 0 | 0 | 0 | 0 | 0 | 0 | 0 | 11 | 0 | 0 | 0 | 0 | 0 | 0 | 0 | 0 | 0 | 0 | 0 | 0 | 0 | 0 |
| 58 | AK13 | This study | 3 | 13 | 64.77 | 147.39 | 0 | 0 | 0 | 0 | 0 | 0 | 0 | 0 | 0 | 0 | 0 | 0 | 0 | 0 | 13 | 0 | 0 | 0 | 0 | 0 | 0 | 0 | 0 | 0 | 0 | 0 | 0 | 0 | 0 |
| 59 | AK14 | This study | 3 | 8 | 64.95 | 147.68 | 0 | 0 | 0 | 0 | 0 | 0 | 0 | 0 | 0 | 0 | 0 | 0 | 0 | 0 | 6 | 0 | 0 | 0 | 0 | 0 | 0 | 0 | 0 | 0 | 2 | 0 | 0 | 0 | 0 |
| 60 | DENA | This study | 3 | 6 | 63.35 | 150.99 | 0 | 0 | 0 | 0 | 0 | 0 | 0 | 0 | 0 | 0 | 0 | 0 | 0 | 0 | 6 | 0 | 0 | 0 | 0 | 0 | 0 | 0 | 0 | 0 | 0 | 0 | 0 | 0 | 0 |
| 61 | JMR | This study | 2 | 8 | 61.38 | 120.60 | 0 | 0 | 0 | 0 | 2 | 0 | 0 | 0 | 0 | 0 | 0 | 0 | 0 | 0 | 4 | 0 | 0 | 0 | 0 | 0 | 0 | 2 | 0 | 0 | 0 | 0 | 0 | 0 | 0 |
| 62 | KAK | This study | 2 | 11 | 61.12 | 118.00 | 0 | 0 | 0 | 0 | 3 | 0 | 0 | 0 | 0 | 0 | 1 | 0 | 0 | 0 | 7 | 0 | 0 | 0 | 0 | 0 | 0 | 0 | 0 | 0 | 0 | 0 | 0 | 0 | 0 |
| 63 | LT | This study | 2 | 12 | 53.63 | 112.84 | 0 | 1 | 0 | 0 | 4 | 0 | 0 | 0 | 0 | 0 | 0 | 0 | 0 | 0 | 5 | 0 | 0 | 0 | 0 | 0 | 0 | 0 | 0 | 0 | 0 | 0 | 0 | 2 | 0 |
| 64 | NAB | This study | 2 | 12 | 60.95 | 123.19 | 0 | 1 | 0 | 0 | 8 | 0 | 0 | 0 | 0 | 0 | 0 | 0 | 0 | 0 | 2 | 0 | 0 | 0 | 0 | 0 | 0 | 0 | 0 | 0 | 0 | 0 | 0 | 1 | 0 |
| 65 | NAVF | This study | 2 | 11 | 61.61 | 125.75 | 0 | 0 | 0 | 0 | 4 | 0 | 0 | 0 | 0 | 0 | 0 | 0 | 0 | 0 | 6 | 0 | 0 | 0 | 0 | 0 | 0 | 1 | 0 | 0 | 0 | 0 | 0 | 0 | 0 |
| 66 | NNPR | This study | 2 | 10 | 61.95 | 127.19 | 0 | 1 | 0 | 0 | 0 | 0 | 0 | 0 | 0 | 0 | 0 | 0 | 0 | 0 | 4 | 0 | 0 | 0 | 0 | 0 | 0 | 3 | 0 | 0 | 0 | 0 | 0 | 1 | 1 |
| 67 | WB01 | This study | 2 | 7 | 59.44 | 112.35 | 0 | 0 | 0 | 0 | 3 | 0 | 0 | 0 | 0 | 0 | 0 | 0 | 0 | 0 | 3 | 0 | 0 | 0 | 0 | 0 | 0 | 0 | 0 | 0 | 0 | 0 | 0 | 1 | 0 |
| 68 | WB02 | This study | 2 | 6 | 60.03 | 112.91 | 0 | 0 | 0 | 0 | 1 | 0 | 0 | 0 | 0 | 0 | 0 | 0 | 0 | 0 | 5 | 0 | 0 | 0 | 0 | 0 | 0 | 0 | 0 | 0 | 0 | 0 | 0 | 0 | 0 |
| 69 | WY | This study | 2 | 7 | 61.93 | 121.74 | 0 | 0 | 0 | 0 | 2 | 0 | 0 | 0 | 0 | 0 | 0 | 0 | 0 | 0 | 5 | 0 | 0 | 0 | 0 | 0 | 0 | 0 | 0 | 0 | 0 | 0 | 0 | 0 | 0 |

**Table S2.** The three cpDNA loci targeted to explore genetic variation across the range of *Larix laricina* including the locus name, primer sequences, and annealing temperature.

| **Locus name** | **Forward primer (F)** | **Reverse primer (R)** | **Annealing temperature (°C)** |
| --- | --- | --- | --- |
|  |  |  |  |
| *Pt26081* | CCCGTATCCAGTATACTTCCA | TGTTTGATTCATTCGTTCAT | 55 |
| *Pt30204* | TCATAGCGGAAGATCCTCTTT | CGGATTGATCCTAACCATACC | 55 |
| *Pt63718* | CACAAAAGGATTTTTTTTCAGTG | CGACGTGAGTAAGAATGGTG | 55 |


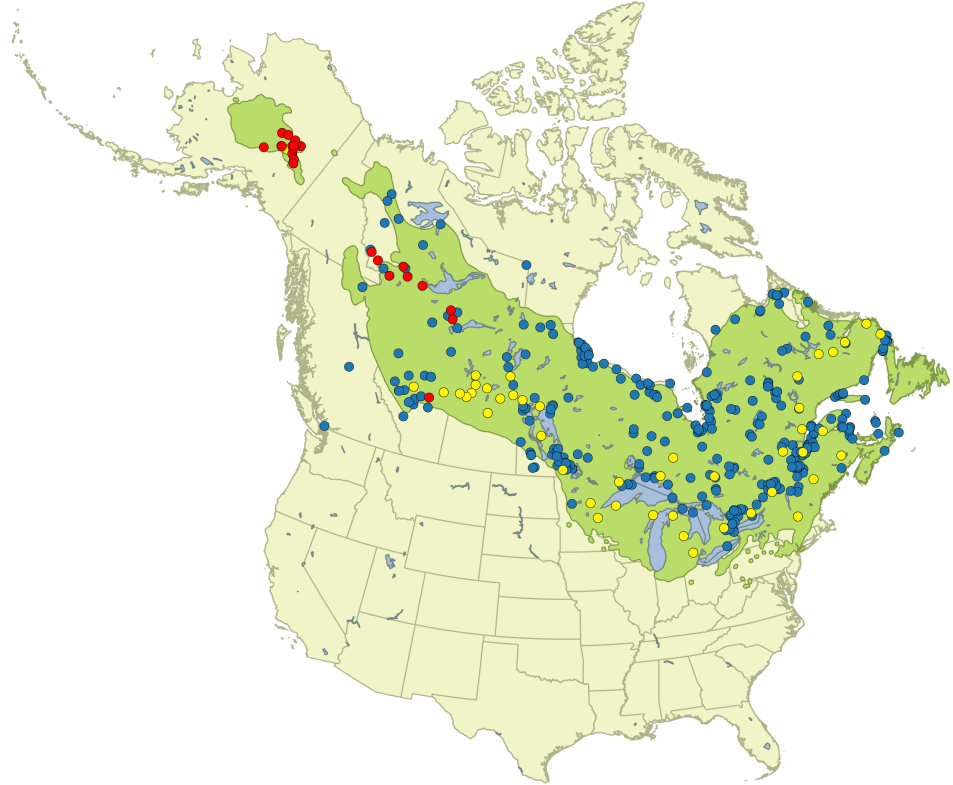


**Fig. S1.** Location of occurrence records for *Larix laricina*. In addition to the 69 locations sampled for population genetics analysis in this study (red circles) and Warren et al. (2016; yellow circles), 370 additional accessions (blue circles) were obtained from the Canadensys (http://www.canadensys.net) database. To prevent overfitting and false inflation of model performance from spatially auto‐correlated occurrences, we implemented the program spThin (Aiello-Lammens, Boria, Radosavljevic, Vilela, & Anderson, 2015) in R 3.3.3 (R Core Team, 2017) to i) reduce each cluster of presence records within a 10 km Euclidian distance to a single point and ii) avoid a spatial sampling bias, by randomly reducing presence points in overrepresented portions of the range. This resulted in a final subset of 341 occurrence to generate our SDMs.

**References**

Aiello-Lammens, M. E., Boria, R. A., Radosavljevic, A., Vilela, B., & Anderson, R. P. (2015). spThin: An R package for spatial thinning of species occurrence records for use in ecological niche models. *Ecography, 38*(5), 541–545. https://doi.org/10.1111/ecog.01132

Barbet-Massin, M., Jiguet, F., Albert, C. H., & Thuiller, W. (2012). Selecting pseudo-absences for species distribution models: How, where and how many? *Methods in Ecology and Evolution, 3*(2), 327–338. https://doi.org/10.1111/j.2041-210X.2011.00172.x

Hijmans, R. J., Cameron, S. E., Parra, J. L., Jones, P. G., & Jarvis, A. (2005). Very high resolution interpolated climate surfaces for global land areas. *International Journal of Climatology, 25*(15), 1965–1978. https://doi.org/10.1002/joc.1276

R Core Team. (2017). *R: A language and environment for statistical computing. R Foundation for statistical computing*. Vienna, Austria: R Core Team.

Walsh, J., Chapman, W., Romanovsky, V., Christensen, J., & Stendel, M. (2008). Global climate model performance over Alaska and Greenland. *Journal of Climate, 21*, 6156–6174. https://doi.org/10.1175/2008jcli2163.1

Warren, E., de Lafontaine, G., Gérardi, S., Senneville, S., Beaulieu, J., Perron, M., . . . Bousquet, J. (2016). Joint inferences from cytoplasmic DNA and fossil data provide evidence for glacial vicariance and contrasted post-glacial dynamics in tamarack, a transcontinental conifer. *Journal of Biogeography, 43*(6), 1227–1241. https://doi.org/10.1111/jbi.12675
